# Cardiovascular disease in breast cancer patients: a nationwide real-world evidence study 2013–20

**DOI:** 10.1093/ehjopen/oeaf043

**Published:** 2025-04-23

**Authors:** Ingrid Engebretsen, Francisco Oteiza, Elisabeth Floberghagen Birkelund, Signe Marie Brandal, Christoffer Bugge, Sigrun Halvorsen

**Affiliations:** Oslo Economics, Klingenberggata 7A, 0161 Oslo, Norway; Oslo Economics, Klingenberggata 7A, 0161 Oslo, Norway; Pfizer AS, Drammensveien 288, 0283 Oslo, Norway; GlaxoSmithKline AS, Drammensveien 288, 0283 Oslo, Norway; Pfizer AS, Drammensveien 288, 0283 Oslo, Norway; Oslo Economics, Klingenberggata 7A, 0161 Oslo, Norway; Department of Cardiology, Oslo University Hospital Ullevål, Kirkeveien 166, 0450 Oslo, Norway; Institute of Clinical Medicine, Faculty of Medicine, University of Oslo, Kirkeveien 166, 0450 Oslo, Norway

**Keywords:** Cardiovascular disease, Female breast cancer, Epidemiology, Cumulative incidence, Mortality, Real-world evidence

## Abstract

**Aims:**

Various measures have been implemented in clinical practice to reduce the risk of cardiovascular complications during breast cancer (BC) treatment. The aim of this study was to investigate whether women diagnosed with BC exhibit a higher incidence of cardiovascular disease (CVD).

**Methods and results:**

Matched cohort study. Using data from the Cancer Registry of Norway and the Norwegian Patient Registry, we created a nationwide cohort of women diagnosed with BC between 2013 and 2020 and age-matched controls (matching ratio 1:10). For BC patients, the index date was the date of their BC diagnosis. For controls, the index date was a random date within the index year of the matched BC patient. For eight selected CVDs, we compared the prevalence before BC diagnosis between cases and controls, as well as the overall incidence, hazard ratios (HRs), and cumulative incidences post BC diagnosis. Follow-up was through 2021. Our study population consisted of 27 526 BC patients and 269 904 matched controls. Among the subset of patients without CVD prior to index, BC patients had significantly increased overall and cumulative risk of pulmonary embolism (HR = 3.00, 95% CI: [2.51–3.59]), atrial fibrillation (1.53 [1.38–1.70]), other cardiac arrhythmias (1.43 [1.27–1.61]), heart failure (1.93 [1.33–2.80]), hypertensive heart disease (1.79 [1.67–1.91]), and heart valve disease (2.02 [1.79–2.27]).

**Conclusion:**

In this contemporary cohort, BC patients still had an increased risk of several CVDs compared to age-matched controls. Further research is needed to determine the causes of this increased risk, but clinicians should be aware and optimize therapy accordingly.

## Graphical Abstract

**Graphical Abstract:**
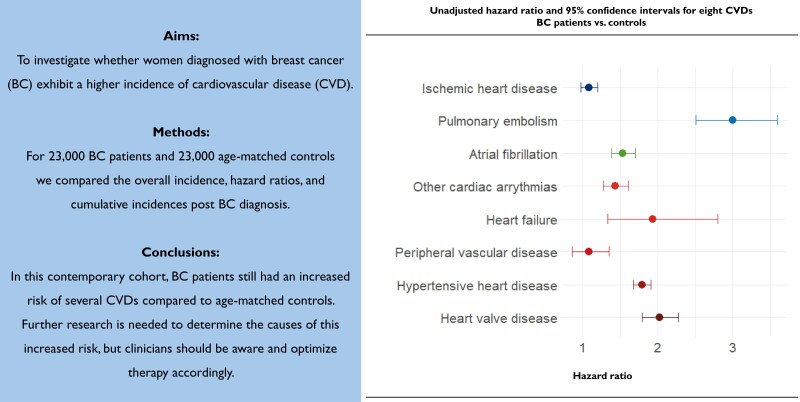

## Introduction

Breast cancer is the most prevalent type of cancer in women.^1^ In 2022, 4247 women were diagnosed with breast cancer (BC) in Norway; this is the highest number since records began in 1953.^2^ Recent progress in the detection and treatment of BC has led to substantial improvement in life expectancy over the past three decades.^3^ As a result, increased attention is now placed on the risk of unintended side effects of cancer therapy, both on the short- and long-term.^4–7^ Besides the increased incidence of cardiovascular disease (CVD) following radiotherapy,^8,9^ others have found that anthracycline and trastuzumab-based therapy is associated with an increased risk of cardiovascular complications.^10–14^ Further, some studies have displayed cardiotoxic effects of cyclin-dependent kinase 4/6 inhibitors.^14–16^ These findings are supported by a retrospective cohort study of all women diagnosed with BC in Denmark between 2003 and 2007, which showed that BC survivors were significantly more likely to develop CVD than the general population.^17^

In 2022, the European Society of Cardiology published guidelines for the prevention of cancer therapy-related cardiovascular toxicity, while highlighting that there is still limited evidence.^14^ Measures have been taken in recent years to reduce the risk of cardiotoxicity during BC treatment. For anthracyclines, national treatment recommendations suggest maximum dose thresholds and more limited use.^18^ To limit cardiac side effects from radiotherapy, active breathing control and hypofractionated regimens have been implemented since 2015.^19^ Other prevention strategies such as electrocardiogram monitoring, administration of angiotensin receptor blocker candesartan, or lifestyle modifications, have shown promising results.^20–23^

The impact of the introduction of these measures, the degree to which these are currently being applied in clinical practice, and how effective they are at limiting toxicity, remains unclear. In this study, we investigated whether recently diagnosed BC patients still exhibit higher incidence of CVD than the general population in a real-world setting. The prevalence and incidence of eight CVD diagnoses among Norwegian female BC patients diagnosed between 2013 and 2020 were compared to those from a control population of women without BC.

## Methods

### Study design

This study was a population-based, matched cohort registry study, linking data from the Cancer Registry of Norway (CRN), the Norwegian Patient Registry (NPR), Statistics Norway and the National Population Register.

### Data sources

The CRN, established in 1953, records all new occurring cancers in Norway as well as selected information regarding treatment and patient status. Information was obtained from the CRN on all women diagnosed with BC from 1953 to 31 December 2020, as well as the patient’s status (alive, dead, and emigrated) up until 31 December 2021.

The National Population Register contains information on the date of birth, address, and date of death for all Norwegian citizens. The National Population Register performed the matching between BC patients and controls, and assigned de-identified, project-specific identifiers to all individuals in our study population.

The NPR holds data on all diagnoses and treatments provided by publicly funded hospitals in Norway, including inpatient and outpatient care. As publicly funded hospitals are responsible for most hospital treatments in Norway, NPR data includes almost all hospital contacts in Norway. Each hospital encounter is recorded with a primary and sometimes a secondary diagnosis using the International Classification of Diseases, Tenth Revision (ICD-10) codes. We obtained data on all hospital contacts related to eight CVDs for all patients in our study sample for the 2008–21 period.

Information about education, income, and region of residence for all individuals in our sample was obtained from Statistics Norway. Individual-level data from each of these registries was linked using project-specific, de-identified individual codes.

### Study population

**Figure 1:**
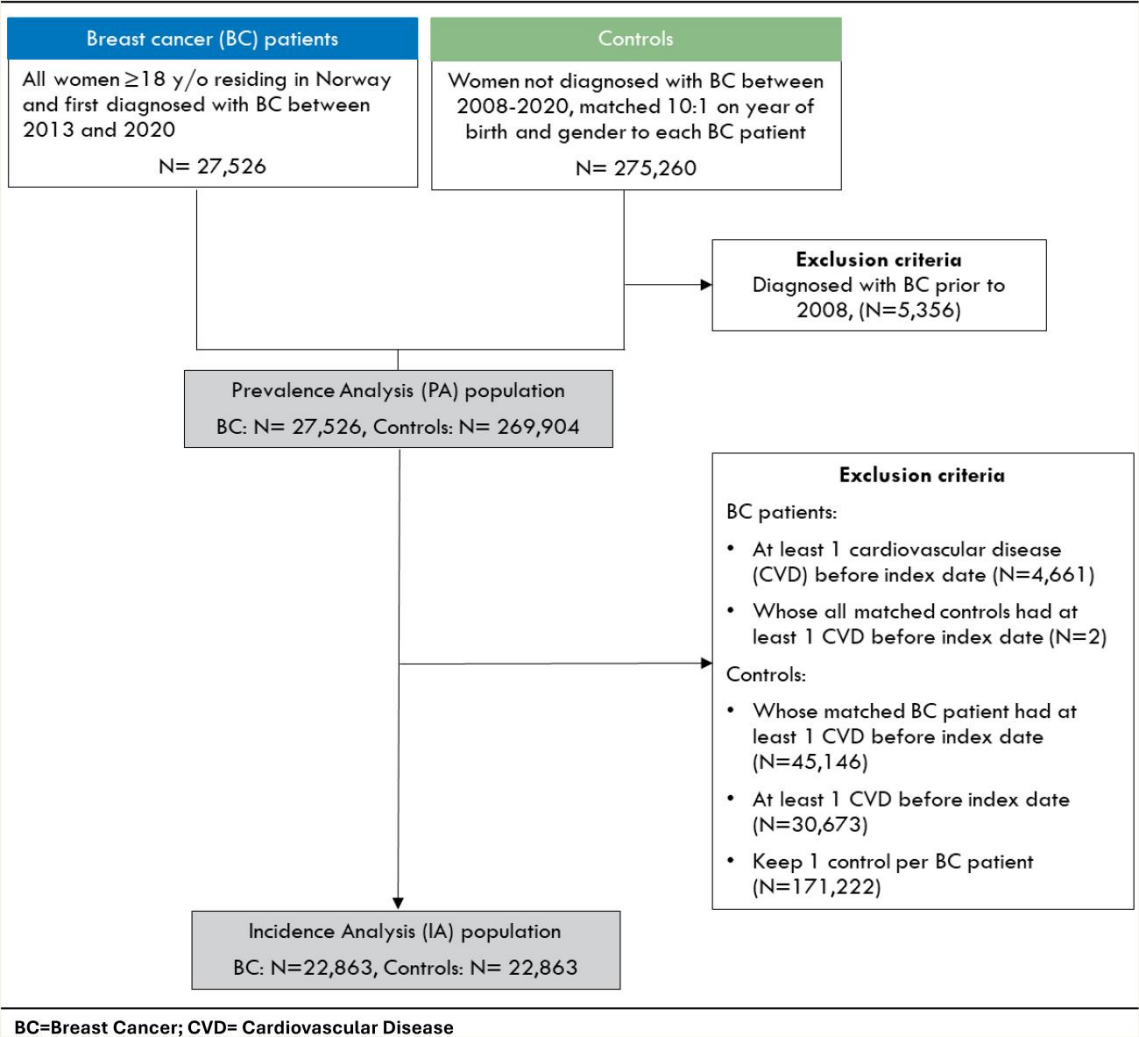
Selection of the study population.

Our initial data extraction consisted of all women ≥ 18 years registered in the CRN with a first diagnosis of BC in all stages in Norway between 1 January 2013 and 31 December 2020. Each of these BC patients was matched to 10 randomly selected controls by the National Population Register. Control individuals were selected to match by gender and year of birth, with the only exclusion criteria being that they could not have been diagnosed with BC between 2008 and 2020 (*Figure 1*). To further ensure that the control group did not include BC survivors, controls diagnosed with BC prior to 2008 were also excluded, using CRN data for 1953–2008.

The index date for BC patients was defined as the date of their first BC diagnosis. The index date for controls was defined as a random date within the same year as the index year for the matched BC patient.^24^ As a result, each BC patient and her matched controls all shared a common year of birth, gender and index year. Patients were followed up until death, emigration, or end of the study period (31 December 2021).

Our prevalence analysis (PA) population thus consists of 27 526 women diagnosed with BC between 2013 and 2020 and 269 904 controls (*Figure 1*). The average number of controls per case in the PA population was 9.8, with a minimum of 6 controls per BC patient.

Cardiovascular disease incidence was estimated based on the incidence analysis (IA) population (*Figure 1*). To create the IA population, all BC patients recorded having at least one of the eight selected CVDs before index date as well as all their matched controls, regardless of their CVD history, were removed from the PA population. All remaining controls with recorded history of the eight included CVDs were then also removed. Two BC patients were left without any controls and were also removed. This led to an unbalanced age distribution between cases and controls, with younger BC patients matched to more controls than older BC patients. Thus, to ensure balance in the age distribution of cases and controls, a single control was randomly selected for each BC patient (1:1 matching). The resulting IA population contained 22 863 BC patients each matched to one control, a total of 45 762 individuals with no history of CVD before their index date (*Figure 1*).

### Primary outcomes

Eight CVDs were identified as relevant outcomes for this study based on discussions with clinical experts and previous studies^17^: ischaemic heart disease, pulmonary embolism, atrial fibrillation, other cardiac arrhythmias, heart failure, peripheral vascular disease, hypertensive heart disease, and heart valve disease. The CVDs were identified based on the disease classification codes used in hospital contacts (ICD-10) (see Supplementary material online, *Table S1*). We considered both primary and secondary diagnoses for all CVDs. A sensitivity analysis was performed using primary diagnoses only.

Prevalence and incidence were estimated individually for each CVD using the PA and IA populations, respectively. As described above, patients identified with previous CVD during the 5-year period before index date, as well as their matched controls regardless of CVD history, were excluded from IA. Patients in the IA population identified as having a CVD during the follow-up period, i.e. after their index date, were defined as incident patients.

### Secondary outcomes

We estimated the cumulative incidence of all-cause mortality. The cumulative incidence was estimated from index date for BC patients and controls in the incident analysis population. Patients who emigrated during follow-up were excluded from these analyses.

### Statistical analysis

Prevalence and incidence were calculated as the number of people with the disease, as well as the incidence proportion among cases and controls. The prevalence of each CVD was estimated among the patients in our PA population using a 5-year period prior to index date. Two-sided *t*-tests were conducted to assess statistical significance, reported in terms of *P*-values. A statistical significance threshold of *P* < 0.05 was applied.

The incidence at any point in time during the follow-up period was estimated for each CVD using the IA population. The length of follow-up for each individual in our sample ranged from one to eight years depending on the index date of each BC patient. Then, the cumulative incidence for each CVD was estimated with hazard ratios (HR) and Kaplan–Meier survival curves. Unadjusted HRs and 95% confidence intervals were estimated by Cox regression. The length of the time at risk was measured as years from index date until the CVD occurred, or until death, emigration, or censoring (end of follow-up). Subgroup analyses of cumulative incidence by age group (<60, 60–69, and >69 years old) at index were also performed.

Statistical differences in cumulative incidence of CVDs and deaths between BC patients and controls were evaluated by log-rank tests. All analyses were conducted using R version 4.1.2 (2021).

### Ethical approval

This project was approved by the Regional Ethics Committee of Norway (REK #219787).

## Results

### Patient characteristics

In both the PA and IA populations, age distributions were similar, by design, for BC patients and controls (*Table 1*). Furthermore, BC and control patients had similar ages, as well as regions of residence, education levels and income distributions at index date. We did, however, not use the variables as matching targets.

**Table 1:** Baseline characteristics of breast cancer (BC) patients and their controls

Population	Prevalent analysis (PA)		Incidence analysis (IA)	
Variable	BC patients	Controls	BC patients	controls
Number of patients, *N*	27 526	269 904	22 863	22 863
Cancer stage at diagnosis, *N* (%)				
Stage I	12 322 (44.8)		10 552 (46.2)	
Stage II	8436 (30.6)		7004 (30.6)	
Stage III	2873 (10.4)		2409 (10.5)	
Stage IV	1100 (4.0)		871 (3.8)	
Unknown	2795 (10.2)		2072 (8.9)	
Age				
Median (IQR)	62 (20)	62 (20)	60 (18)	60 (18)
<60 years, *N* (%)	11 748 (42.7)	116 933 (43.3)	11 116 (48.6)	11 116 (48.6)
60–69 years, *N* (%)	7616 (27.7)	74 459 (27.6)	6530 (28.6)	6530 (28.6)
70+ years, *N* (%)	8162 (29.7)	78 512 (29.1)	5217 (22.8)	5217 (22.8)
Region of residence, *N* (%)				
Central	4573 (16.6)	47 129 (17.5)	3764 (16.5)	4015 (17.6)
North	2185 (7.9)	24 152 (8.9)	1778 (7.8)	1995 (8.7)
South–East	13 712 (49.8)	128 496 (47.6)	11 515 (50.4)	10 924 (47.8)
West	6925 (25.2)	66 576 (24.7)	5680 (24.8)	5575 (24.4)
Unknown	131 (0.5)	3551 (1.3)	126 (0.6)	354 (1.6)
Education level, *N* (%)				
High school or lower	17 474 (63.5)	177 783 (65.9)	13 908 (60.8)	14 460 (63.3)
Higher education	9797 (35.6)	87 890 (32.6)	8725 (38.2)	8002 (35.0)
Unknown	255 (0.9)	4231 (1.6)	230 (1.0)	401 (1.8)
Household income in Norwegian krone				
Median income (IQR)	569 261 (480 495)	558 784 (482 967)	604 860 (483 788)	592 260 (493 271)

### Prevalence of cardiovascular disease prior to index

During the 5 years prior to their index date, there were no significant differences in the prevalence of heart failure, peripheral vascular disease, and heart valve disease between BC patients and controls (*P* > 0.05) (*Table 2*). Pulmonary embolism, atrial fibrillation, other cardiac arrhythmias, and hypertensive heart disease were more prevalent among BC patients, while ischaemic heart disease was more prevalent among controls.

**Table 2:** Prevalence of selected cardiovascular diseases (CVD), hypertensive heart disease, and heart valve disease among breast cancer patients and controls during the 5 years prior to index date (PA population)

Condition	Breast cancer patients, *N* (%)	Controls, *N* (%)	*P*-value (BC vs. Controls)
Ischaemic heart disease	1148 (4.2)	12 089 (4.5)	0.019
Pulmonary embolism	178 (0.7)	1301 (0.5)	<0.001
Atrial fibrillation	1182 (4.3)	10 164 (3.8)	<0.001
Other cardiac arrhythmias	691 (2.5)	5999 (2.2)	0.002
Heart failure	67 (0.2)	667 (0.2)	0.956
Peripheral vascular disease	187 (0.7)	1712 (0.6)	0.393
Hypertensive heart disease	2747 (10.0)	25 673 (9.6)	0.020
Heart valve disease	693 (2.5)	6384 (2.4)	0.119

The use of primary hospital diagnoses only for the identification of CVDs, revealed the same pattern of results. The only exception was the difference in the prevalence of ischaemic heart disease, which no longer was statistically significant (see Supplementary material online, *Table S2*).

### Overall incidence of cardiovascular disease after index

After index date, BC patients showed significantly higher incidence of pulmonary embolism, atrial fibrillation, other cardiac arrhythmias, heart failure, hypertensive heart disease, and heart valve disease, compared to controls (*Table 3*). There was no significant difference in the incidence of ischaemic heart disease and peripheral vascular disease. Within the heart valve disease condition, mitral valve insufficiency was the specific disease with the largest incidence difference after index between BC patients and controls (1.7% vs. 0.6%) (see Supplementary material online, *Table S4*).

**Table 3:** Incidence of cardiovascular disease (CVD) among breast cancer (BC) patients and controls at any point after index date (%)

Condition	BC patients, *N* (%)	Controls, *N* (%)	*P*-value (BC ≠ Control)
Ischaemic heart disease	672 (2.9)	687 (3.0)	0.700
Pulmonary embolism	456 (2.0)	167 (0.7)	<0.001
Atrial fibrillation	873 (3.8)	635 (2.8)	<0.001
Other cardiac arrhythmias	626 (2.7)	493 (2.2)	<0.001
Heart failure	78 (0.3)	44 (0.2)	0.003
Peripheral vascular disease	152 (0.7)	157 (0.7)	0.819
Hypertensive heart disease	2347 (10.3)	1483 (6.5)	<0.001
Heart valve disease	753 (3.3)	412 (1.8)	<0.001

In the sensitivity analysis based on primary diagnoses only, the same result was found for all CVDs except for hypertensive heart disease, which ceased to be significantly different (*P* = 0.10) (see Supplementary material online, *Table S3*).

### Hazard ratios and cumulative incidence of cardiovascular disease after index

The unadjusted HR among BC patients compared to controls was significantly higher for pulmonary embolism, atrial fibrillation, other cardiac arrhythmias, heart failure, hypertensive heart disease, and heart valve disease (*Table 4*). The unadjusted HR was largest for pulmonary embolism and heart failure. There was no significant difference for ischaemic heart disease and peripheral vascular disease.

**Table 4:** Unadjusted hazard ratio (HR) and 95% CI for developing cardiovascular diseases among breast cancer (BC) patients compared to controls

Condition	Unadjusted HR [95% CI] BC patients vs. controls
Ischaemic heart disease	1.08 [0.97–1.20]
Pulmonary embolism	3.00 [2.51–3.59]
Atrial fibrillation	1.53 [1.38–1.70]
Other cardiac arrhythmias	1.43 [1.27–1.61]
Heart failure	1.93 [1.33–2.80]
Peripheral vascular disease	1.08 [0.86–1.35]
Hypertensive heart disease	1.79 [1.67–1.91]
Heart valve disease	2.02 [1.79–2.27]

**Figure 2:**
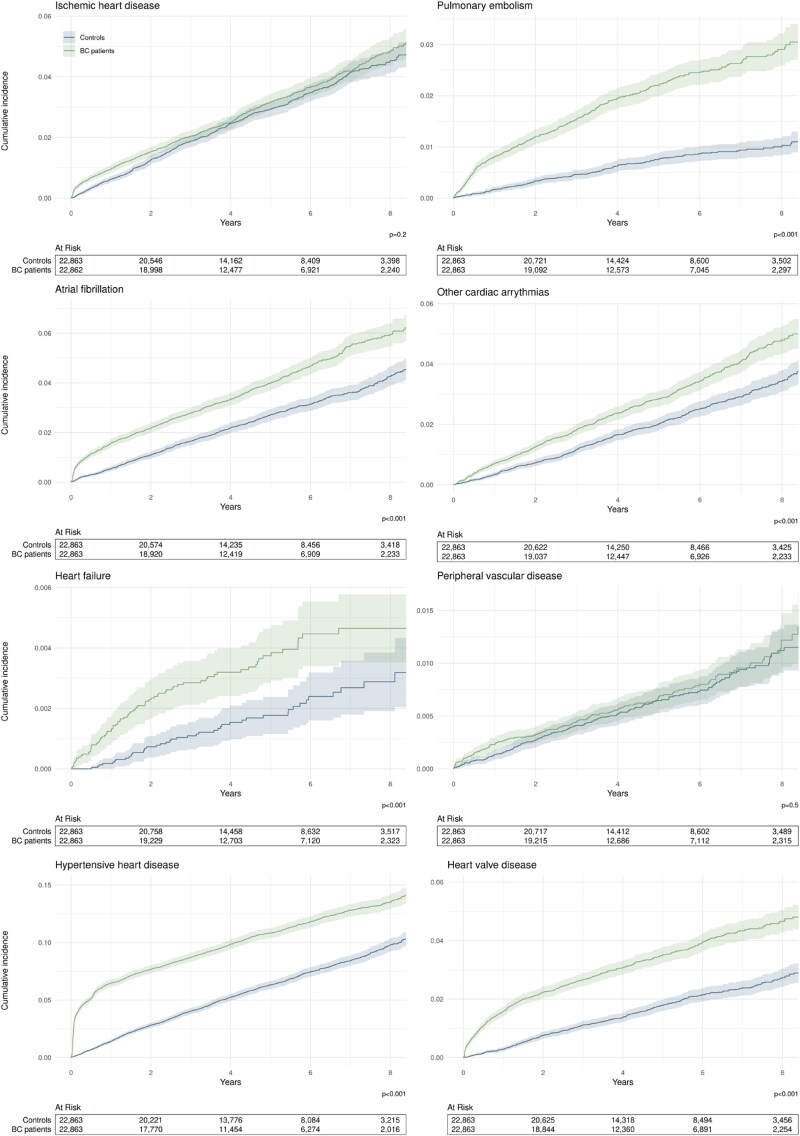
Cumulative incidence of specific cardiovascular diseases since index date.

Similarly, in Kaplan–Meier survival analyses, the cumulative incidences of all the included CVDs except ischaemic heart disease and peripheral vascular disease were significantly higher among BC patients compared to controls (*P* < 0.001) (*Figure 2*). Cumulative incidence plots showed different patterns by disease (*Figure 2*). In the case of hypertensive heart disease, atrial fibrillation, and heart valve disease, incidence among BC patients increased sharply during the first year following index date. However, after this initial divergence, incidence for both BC patients and controls appeared to be similar. On the other hand, for pulmonary embolism, heart failure, and other cardiac arrhythmias, the gap in cumulative incidence appeared to widen more gradually over time, suggesting persistently higher incidence for BC patients. In a sensitivity analysis, we plotted the cumulative incidence of CVD by age group (<60, 60–69, and >69 years) to assess whether these findings were dependent on the patient’s age at index (see Supplementary material online, *Figure S1*). For ischaemic heart disease, there were no significant differences in cumulative incidence between cases and controls in any of the age groups. For pulmonary embolism, cumulative incidence was higher for BC patients and the difference increased over time in all age groups. For atrial fibrillation, cumulative incidence patterns were different for different age groups. For patients <60 years and 60–69 years at index, the gap in cumulative incidence between BC patients and controls seemed to widen over time. For patients >69 years of age, the incidence of atrial fibrillation was higher among BC patients shortly after index date, but then converged to the same incidence rates as for controls. For other cardiac arrhythmias, heart failure, peripheral vascular disease, and heart valve disease, the difference between BC patients and controls was most evident in the youngest age group.

### Cumulative incidence of all-cause mortality

The cumulative incidence of all-cause mortality in the incident analysis population was higher among BC patients compared to controls (*P* < 0.001) (see Supplementary material online, *Figure S2*). The 2-year cumulative incidence of death was 5.4% (95% CI: 5.1–5.7%) for BC patients and 2.8% (95% CI: 2.5–3.0%) among controls. The 6-year cumulative incidence of death was 14.8% (95% CI: 14.2–15.3%) among BC patients, and 7.9% (95% CI 7.5–8.3%) among controls.

In total, 2794 BC patients and 1556 controls in the incident analysis population died during follow-up. Among the 2794 BC patients who died, 61.8% (*N* = 1729) died of cancer, 17.9% (*N* = 499) died from other causes than cancer, while cause of death was not reported for 26.6% (*N* = 742). We were not able to distinguish CVD deaths from other causes than cancer, and we did not have information on cause-specific mortality for the control group.

## Discussion

In this nationwide cohort study, we found that women diagnosed with BC between 2013 and 2020 in Norway had a higher cumulative risk of developing pulmonary embolism, atrial fibrillation, other cardiac arrhythmias, heart failure, hypertensive heart disease, and heart valve disease after their diagnosis. No difference was observed in the incidence of ischaemic heart disease and peripheral vascular disease between BC patients and controls.

At the time of their first BC diagnosis, the cohort of BC patients exhibited similar observable characteristics as their matched controls without BC, including age, education, income, and region of residence. Despite these similarities, we found a slightly higher prevalence of CVD among BC patients prior to their BC diagnosis. There are several possible factors such as lifestyle risk factors and genetic susceptibility that may influence a patient’s probability of suffering from both CVD and BC.^14^

Since we were not able to control for all the possible risk factors influencing both the risk of BC and CVD, the incidence of CVD after a first BC diagnosis was measured only on a subset of patients that had no history of CVD prior to their index date. In this subset of patients (the IA population), we found statistically significant and clinically meaningful differences in both overall incidence proportions and cumulative incidence. Overall incidence was higher for BC patients for most of the studied CVDs, except for ischaemic heart disease and peripheral vascular disease. Breast cancer patients were more likely than controls to be diagnosed with hypertensive heart disease, pulmonary embolism, atrial fibrillation, other cardiac arrhythmias, heart valve disease, and heart failure. The largest overall risk difference was observed for heart failure and pulmonary embolism. Similarly, we found that BC patients had a higher hazard and cumulative incidence of developing all of the included CVDs, except for ischaemic heart disease and peripheral vascular disease. For pulmonary embolism, heart failure, and other cardiac arrhythmias, the gap in cumulative incidence appeared to widen more gradually over time, suggesting persistently higher incidence for BC patients. For atrial fibrillation, hypertensive heart disease, and heart valve disease, BC patients exhibited higher incidence shortly after BC diagnoses, but incidence rates converged to those of the controls over time.

There may be several factors influencing the increased incidence of CVDs among BC patients. Firstly, some cancer-therapies may have cardiotoxic side effects, such as radiotherapy,^8,9^ anthracyclines, and trastuzumab-based therapies.^10–14^ Secondly, other factors such as age, lifestyle, and genetics may all contribute to the increased risk of developing CVD. In this study, we controlled age by matching BC patients to control patients born on the same year, but did not account for other factors such as smoking or obesity due to lack of information. Third, BC patients in active BC treatment are more likely to be closely monitored than the matched controls. Closer follow-up of BC patients in the health care system may lead to earlier detection and diagnosis of asymptomatic and symptomatic CVDs. The use of echocardiogram to screen and follow left ventricular function during chemotherapy could be one of the explanations for the increased risk of heart valve disease in BC patients.

The strengths of this study lie in its long-term follow-up of a nationwide cohort of BC patients in Norway, along with a control group of patients without BC. Additionally, the Norwegian healthcare system, which is tax-funded and provides access to all citizens, ensures that the registries include data from all individuals residing in the country. This inclusiveness enables precise estimation of the prevalence and incidence of CVDs, as only a small number of patients seek care in the private sector.

Some important limitations need to be addressed. First, we did not have information on several factors that might affect both the risk of BC and CVD, such as hormonal replacement therapy, smoking, and other lifestyle risk factors. Neither did we have data on genetic alterations in BC which may affect CV risk. Second, we were not able to evaluate to what extent the increased risk of CVD in BC patients was caused by earlier detection due to closer follow-up in the health care system. However, cumulative incidence plots revealed persistently higher cumulative incidence of pulmonary embolism, atrial fibrillation, heart failure, and other cardiac arrhythmias in BC patients, arguing against this hypothesis. Third, the risk of developing one CVD may affect the risk of developing other CVDs. We cannot identify the precise mechanisms by which patients, directly or indirectly, develop each of the conditions studied. Fourth, we had limited data on causes of death, and we were therefore not able to distinguish deaths caused by CVD from deaths from other causes. Fifth, some patients may have a CVD that is diagnosed and treated in the primary sector only, and these would not be captured in this study. The incidence of some CVDs, such as hypertension, may therefore be underestimated. Lastly, the Norwegian health care system is financed by taxation, with universal access to all residents. The use of private medical insurance is limited in Norway. The generalizability of our results to countries with different health care systems may be limited.

There are a limited number of registry-based studies that have investigated CVD incidence among BC patients. A study conducted in Denmark on BC patients diagnosed between 2003 and 2007 found statistically significant differences in CVD incidence after BC diagnosis for heart failure, hypertensive heart disease, phlebitis and pulmonary embolism, compared to a set of controls.^17^ Their estimated differences in incidence were somewhat smaller than we document in this study. Given that their study was based on a cohort of patients diagnosed a decade earlier than those in our study, we would expect larger differences in CVD incidence in their study compared to ours. Their analysis of incidence was conducted by removing only CVD-prevalent patients from the sample, probably partly explaining the smaller differences found in the Danish study. In this Norwegian study, we also removed all controls, regardless of their CVD-status, whenever their matched BC patient was CVD-prevalent. This was done to avoid keeping controls with no BC patient in our IA population, which might lead to an under-estimation of the true difference in CVD incidence.

A study from the US describing the prevalence of pre-existing CVDs in patients with HR+/HER2 metastatic BC found a high prevalence of CVD, with 61% having at least one CVD at time of BC diagnosis.^25^ This was mainly driven by the high prevalence of hypertension in the sample (51%). The prevalence of hypertension is higher in the US general population than in Norway. It is estimated that approximately half of the US population suffer from hypertension,^26^ while the prevalence in Norway is estimated to 25% for women aged 40–79 years.^27^ Moreover, the US study only included metastatic BC patients, while the present study included BC patients diagnosed at all stages.

A matched cohort study from the US assessing cardiovascular incidence among older BC survivors found that BC patients had a slightly higher prevalence of hypertension at diagnosis, compared to matched controls.^24^ However, they found a slightly lower prevalence of other pre-existing CVDs among BC patients compared to controls. In line with our findings, they found an increased risk of arrhythmias, heart failure, and heart valve disease among BC patients. Further, they found that BC patients had a lower risk of myocardial infarction, stroke, and peripheral vascular disease after diagnosis, compared to matched controls. Another matched cohort study from the US found increased risk of CVD (ischaemic heart disease, stroke, and cardiomyopathy/heart failure) among BC patients,^28^ while a matched cohort study from Canada documented increased risk of heart failure.^29^ Differences in study populations and health care systems, along with the use of private insurance data covering only a subsample of BC patients,^24,28^ makes comparisons challenging. Increased risk of CVD among BC patients has also been documented in non-registry-based studies.^30,31^

While this study does not aim to identify the causal mechanism driving the increased incidence of CVD observed among BC patients, cumulative incidence graphs may provide an indication about the possible drivers. If the increased incidence is caused by earlier detection due to closer follow-up for BC patients, we would expect to see an initial spike in the BC group, but controls would catch up with the incidence rates of the BC patients over time. In the cumulative incidence plots presented in this study, we do not see the eventual catch up in the control group, although we do not have data throughout the patients’ lifetime. Further research is needed to disentangle the contribution of each of these factors to CVD incidence among BC patients. Irrespective of the causal mechanisms, clinicians should be aware of the increased risk of CVD in BC patients and optimize therapy accordingly for better outcomes for BC patients.

## Conclusion

Women diagnosed with BC between 2013 and 2020 in Norway had a higher risk of developing pulmonary embolism, atrial fibrillation, other cardiac arrhythmias, heart failure, peripheral vascular disease, hypertensive heart disease, and heart valve disease after their diagnosis compared to age-matched controls. No significant difference was observed for ischaemic heart disease. More research is needed to determine the causes of this increased risk of CVD, which may be multiple and related both to common risk factors, cardiotoxicity of cancer therapy, and earlier detection. However, clinicians should be aware of the increased risk and optimize therapy accordingly for better treatment outcomes for BC patients.

## Lead author biography


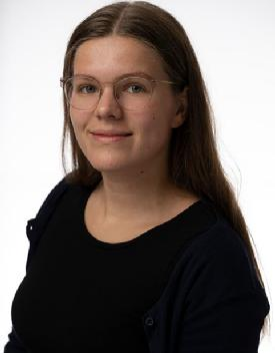


Ingrid Engebretsen holds a master’s degree in economics from the University of Oslo, with a specialization in data analysis and statistics. She is currently a Ph.D. student at The Institute of Behavioral Medicine within The Faculty of Medicine at the same university. She is also employed by Oslo Economics, where she primarily works with registry research. Her fields of interest include atherosclerotic cardiovascular diseases, medication adherence, health economic analyses, and statistics.

## Supplementary Material

oeaf043_Supplementary_Data

## Data Availability

Data cannot be shared for ethical/privacy reasons.
