# Supplementary material for: Cardiovascular disease in breast cancer patients: a nationwide real-world evidence study 2013–20
Source: Eur Heart J Open. 2025 Apr 23;5(3):oeaf043. doi: 10.1093/ehjopen/oeaf043 (PMC12042752; doi:10.1093/ehjopen/oeaf043)
Supplement: oeaf043_Supplementary_Data [file oeaf043_supplementary_data.docx]

## Supplementary material

Supplementary table 1: Identification of cardiovascular diseases using health registry records

| **Condition** | **Definition** | **Reimbursement codes included in definition** |
| --- | --- | --- |
| Hypertensive heart disease | One hospital contact (NPR) with relevant ICD-10 code | Essential (primary) hypertension (ICD-10 I10)  Hypertensive heart disease without (congestive) heart failure (ICD-10 I119)  Hypertensive renal disease (ICD-10 I12),  Hypertensive heart and renal disease with renal failure (ICD-10 I131) Hypertensive heart and renal disease, unspecified (ICD-10 I139) Secondary hypertension (ICD-10 I15) |
| Ischemic heart disease | One hospital contact (NPR) with relevant ICD-10 code. | Angina pectoris (ICD-10 I20)  Acute myocardial infarction (ICD-10 I21)  Subsequent myocardial infarction (ICD-10 I22)  Chronic ischemic heart disease (ICD- 10 I25) |
| Pulmonary embolism | One hospital contact (NPR) with relevant ICD-10 code. | Pulmonary embolism (ICD-10 I26) |
| Atrial fibrillation | One hospital contact (NPR) with relevant ICD-10 code | Atrial fibrillation and flutter (ICD-10 I48) |
| Other cardiac arrythmias | One hospital contact (NPR) with relevant ICD-10 code. | Other cardiac arrhythmias (ICD-10 I49) |
| Heart failure | One hospital contact with relevant ICD-10 code | Heart failure (ICD-10 I50) Dilated cardiomyopathy (ICD-10 I420) Cardiomyopathy due to drugs and other external agents (ICD-10 I427) Cardiomyopathy, unspecified (ICD-10 I429) Hypertensive heart disease with (congestive) heart failure (I110) Hypertensive heart and renal disease with (congestive) heart failure (ICD-10 I130) Hypertensive heart and renal disease with both (congestive) heart failure and renal failure (ICD-10 I132) |
| Heart valve disease | One hospital contact (NPR) with relevant ICD-10 code | Mitral (valve) insufficiency (ICD-10 I340)  Mitral (valve) prolapse (ICD-10 I341)  Nonrheumatic mitral (valve) stenosis (ICD-10 I342)  Nonrheumatic mitral valve disorder, unspecified (ICD-10 I349)  Aortic (valve) stenosis (ICD-10 I350) Aortic (valve) insufficiency (ICD-10 I351)  Aortic valve disorder, unspecified (ICD-10 I359) |
| Peripheral vascular disease | One hospital contact (NPR) with relevant ICD-10 code | Aortic aneurysm and dissection (ICD-10 I71) Peripheral vascular disease, unspecified (ICD-10 I739) Gangrene, not elsewhere classified (ICD-10 R02) |

Supplementary table 2: Prevalence of cardiovascular disease (CVD) among breast cancer patients and controls five years before index date – restricted to primary diagnosis for hospital contacts only.

| **Condition** | **Breast cancer patients N (%)** | **Controls, N (%)** | **P-value**  **(BC ≠ Control)** |
| --- | --- | --- | --- |
| Ischemic heart disease | 849 (3.1) | 8,770 (3.2) | 0.146 |
| Pulmonary embolism | 131 (0.5) | 974 (0.4) | 0.003 |
| Atrial fibrillation | 731 (2.7) | 6,098 (2.3) | <0.001 |
| Other cardiac arrythmias | 653 (2.5) | 5,488 (2.0) | <0.001 |
| Heart failure | 41 (0.2) | 418 (0.2) | 0.875 |
| Peripheral vascular disease | 146 (0.5) | 1,366 (0.5) | 0.620 |
| Hypertensive heart disease | 674 (2.5) | 6,294 (2.3) | 0.231 |
| Heart valve disease | 532 (1.9) | 4,825 (1.8) | 0.089 |

Supplementary table 3: Incidence of cardiovascular disease (CVD) among breast cancer patients and controls at any point after index date (%)– restricted to primary diagnosis for hospital contacts only.

| **Condition** | **BC patients, N (%)** | **Controls, N (%)** | **P-value**  **(BC ≠ Controls)** |
| --- | --- | --- | --- |
| Ischemic heart disease | 593 (2.4) | 629 (2.6) | 0.311 |
| Pulmonary embolism | 341 (1.4) | 148 (0.6) | <0.001 |
| Atrial fibrillation | 558 (2.3) | 442 (1.8) | <0.001 |
| Other cardiac arrythmias | 611 (2.5) | 487 (2.0) | <0.001 |
| Heart failure | 71 (0.3) | 36 (0.2) | 0.001 |
| Peripheral vascular disease | 145 (0.6) | 126 (0.5) | 0.273 |
| Hypertensive heart disease | 547 (2.2) | 493 (2.0) | 0.097 |
| Heart valve disease | 694 (2.8) | 330 (1.4) | <0.001 |

Supplementary table 4: Prevalence and incidence of specific diagnostic codes (ICD-10) within the heart valve disease condition

|  | **Prevalence before index, N (%)** | | **Incidence after index, N (%)** | |
| --- | --- | --- | --- | --- |
| **Type of valvular disease** | **Breast cancer patients** | **Controls** | **Breast cancer patients** | **Controls** |
| Mitral valve insufficiency (ICD-10 I340) | 252 (0.9) | 2168 (0.8) | 392 (1.7) | 140 (0.6) |
| Mitral valve prolapse (ICD-10 I341) | 22 (0.1) | 183 (0.1) | 16 (0.1) | 7 (0.0) |
| Nonrheumatic mitral valve stenosis (ICD-10 I342) | 12 (0.0) | 132 (0.0) | 9 (0.0) | 7 (0.0) |
| Nonrheumatic mitral valve disorder, unspecified (ICD-10 I349) | 16 (0.1) | 116 (0.0) | 3 (0.0) | 2 (0.0) |
| Aortic valve stenosis (ICD-10 I350) | 291 (1.1) | 3050 (1.1) | 211 (0.9) | 193 (0.8) |
| Aortic valve insufficiency (ICD-10 I351) | 227 (0.8) | 1870 (0.7) | 205 (0.9) | 115 (0.5) |
| Aortic valve disorder, unspecified (ICD-10 I359) | 35 (0.1) | 379 (0.1) | 23 (0.1) | 8 (0.0) |

Supplementary figure 1: Cumulative incidence of cardiovascular disease since index date, by BC status and age group

| 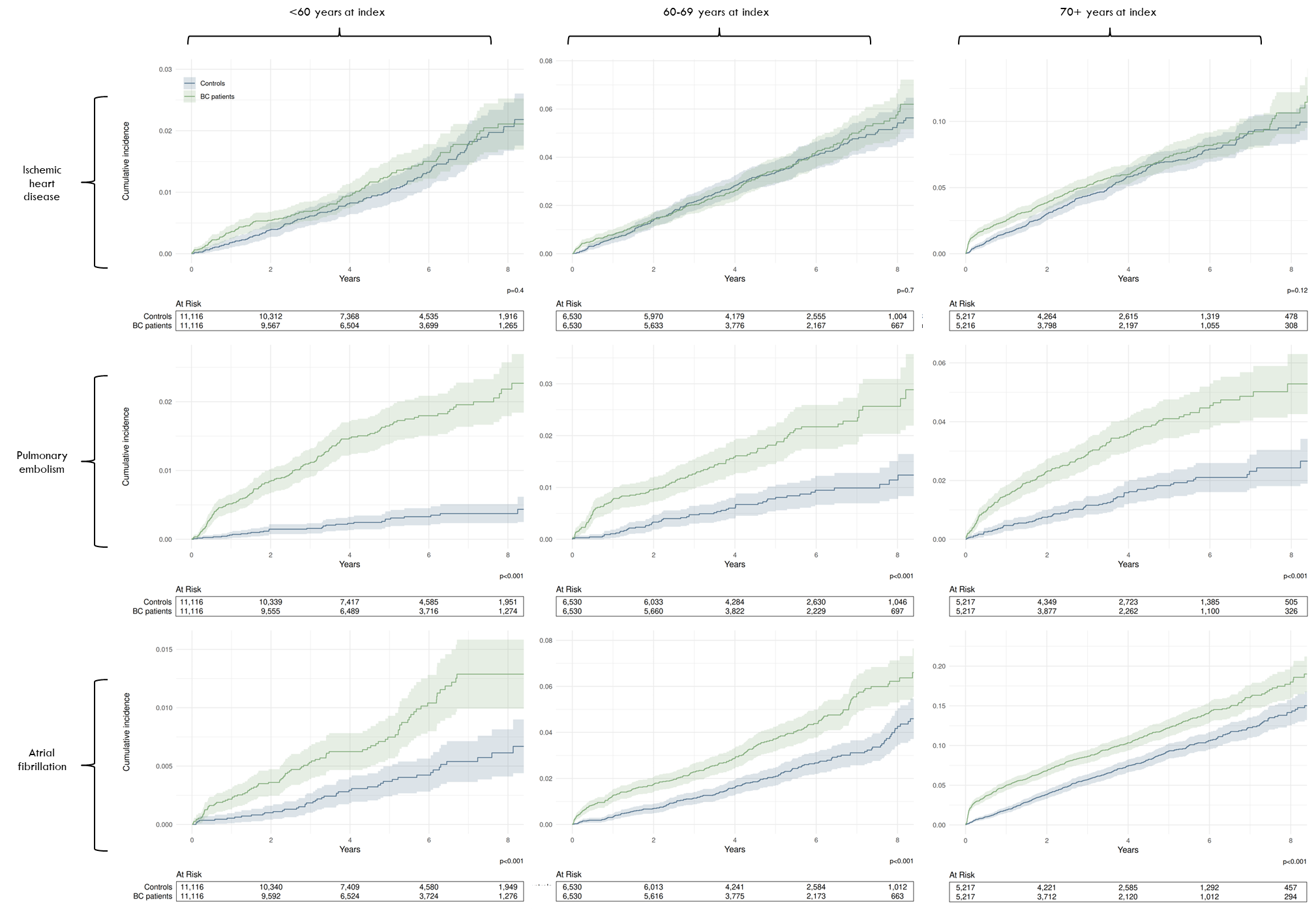 |
| --- |
| 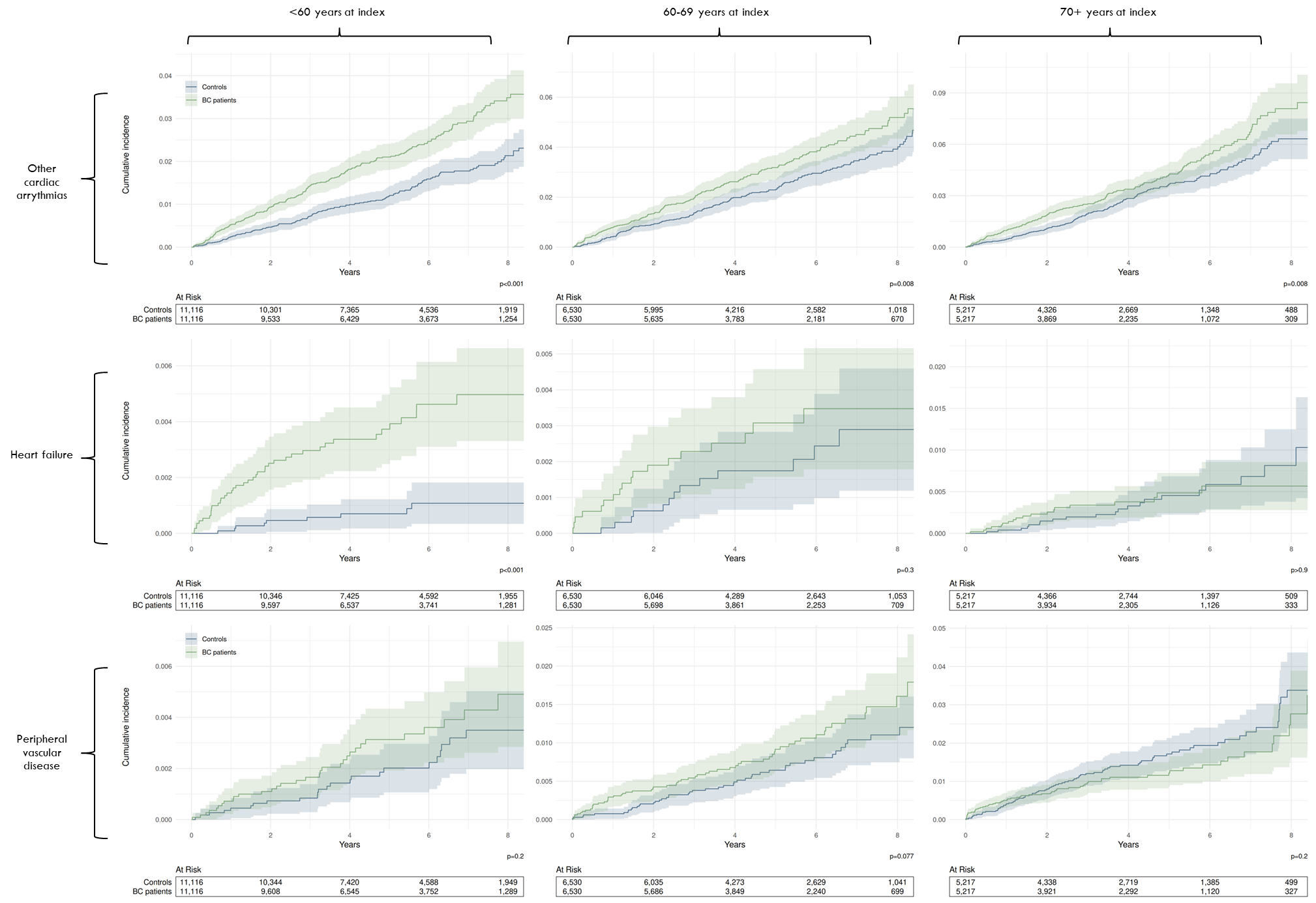 |
| 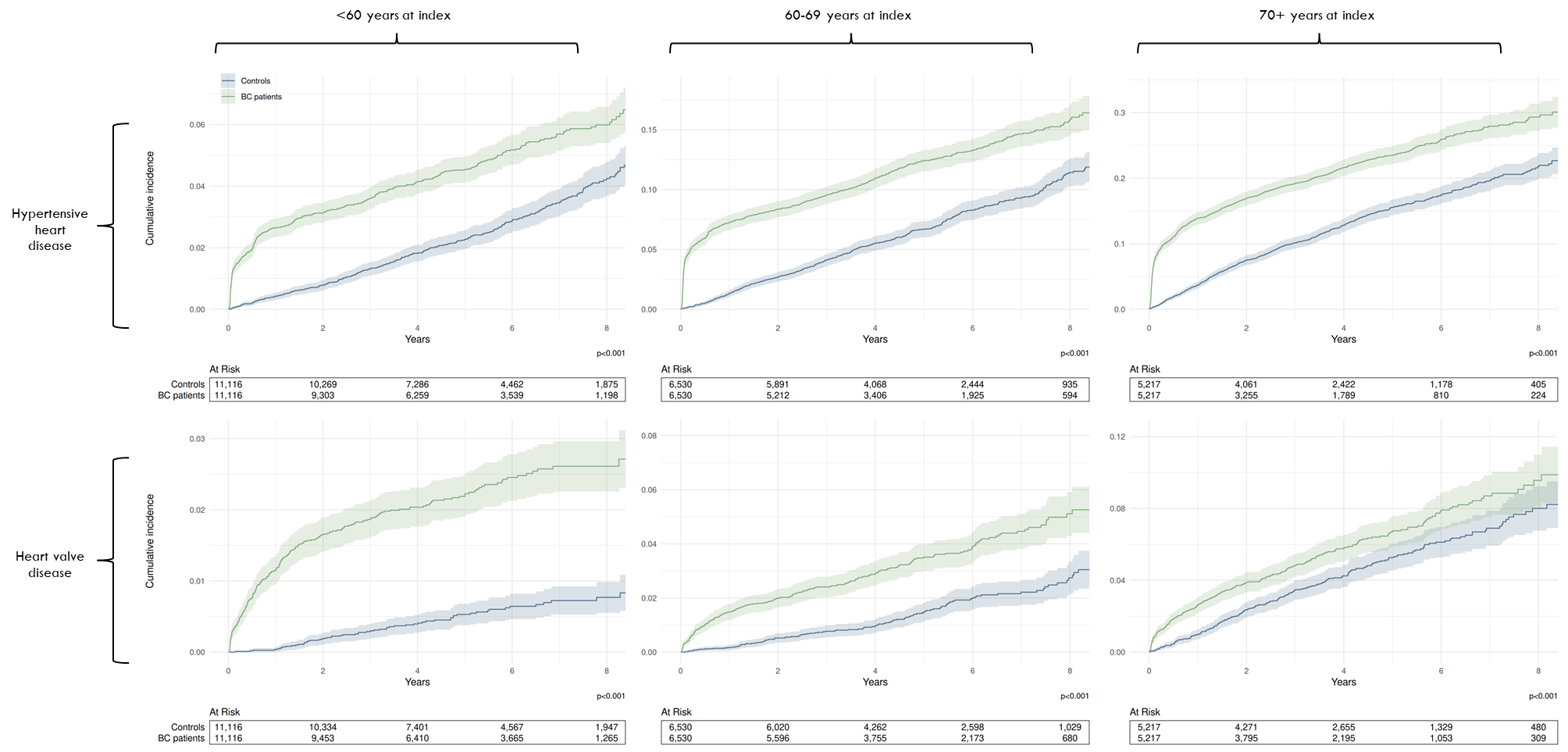 |

Supplementary figure 2: Cumulative incidence all-cause mortality in breast cancer patients and controls free of CVD at the time of breast cancer diagnosis

| 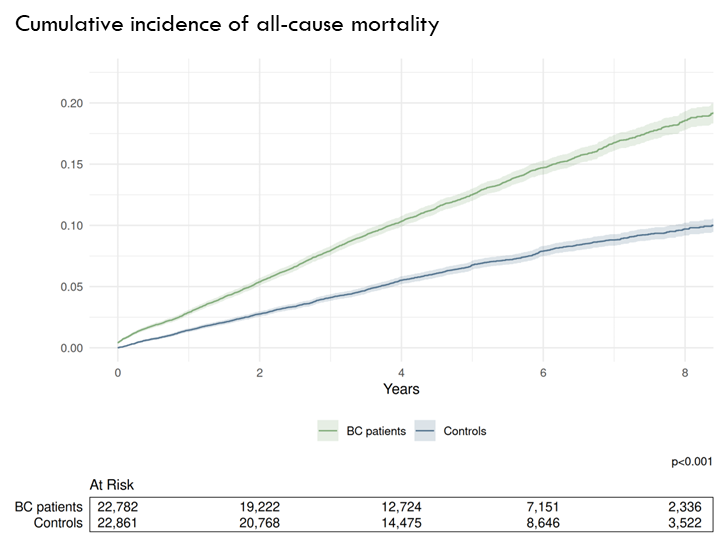 |
| --- |
